# Systemic immune-inflammatory index predicts all-cause and cardiovascular mortality in metabolic syndrome: A prospective cohort study using NHANES data

**DOI:** 10.1016/j.clinsp.2026.100919

**Published:** 2026-04-16

**Authors:** Yao Sun, Shuguang Yang, Zengli Xiao, Youzhong An, Huiying Zhao

**Affiliations:** a Department of Critical Care Medicine, Peking University People's Hospital, No.11 Xizhimen South Street, Xicheng District, Beijing, PR China; b Department of Critical Care Medicine, Sichuan Academy of Medical Sciences & Sichuan Provincial People’s Hospital, University of Electronic Science and Technology of China, Chengdu, PR China

**Keywords:** Systemic immune-inflammatory index (SII), Metabolic syndrome, National health and nutrition examination survey (NHANES), All mortality, Cardiovascular mortality

## Abstract

**Background:**

This study aims to evaluate the association between SII and the prevalence of MetS in the female population of the United States, utilizing data from the National Health and Nutrition Examination Survey (NHANES) 2013–2018.

**Methods:**

The study analyzed data from 6140 participants. The association between SII and MetS was investigated using both univariate and multivariable analyses. Additionally, the relationship between SII and all-cause as well as cardiovascular mortality was assessed through Cox proportional hazards regression models.

**Results:**

MetS was prevalent in 37.52% of the study population. Participants diagnosed with MetS showed higher SII scores compared to those without MetS. There was a nonlinear “J”-shaped dose-response relationship between increased SII levels and increased all-cause mortality in the MetS population. The same trend of relationship between SII levels and cardiovascular mortality was observed in people aged 60-years and older in the MetS population.

**Conclusion:**

The results of this study suggest that there is a significant association between elevated SII and the presence of MetS, and that systemic inflammation indicated by SII may contribute to the development and progression of MetS and its associated complications. Further studies are warranted to elucidate the underlying mechanisms between SII and MetS and to evaluate the clinical utility of SII in risk stratification and management strategies.

## Highlights

• SII is a cost-effective indicator for Metabolic Syndrome (MetS).
• MetS patients exhibit significantly higher SII scores than healthy peers.
• High SII predicts increased all-cause and CVD mortality in MetS.
• A J-shaped relationship exists between SII and MetS mortality risks.
• SII mortality prediction is strongest in MetS patients aged ≥60.

## Introduction

The Systemic Immune-Inflammatory Index (SII) is a novel and stable marker of inflammation, capable of reflecting both the local immune response and the systemic inflammatory state. SII is calculated as the platelet count multiplied by the neutrophil count, divided by the lymphocyte count.^1^ This composite parameter, which integrates peripheral platelet, neutrophil, and lymphocyte counts, offers a more comprehensive assessment of the body's inflammatory status than individual inflammatory markers. Research has demonstrated that SII possesses superior predictive value for major cardiovascular events compared to traditional risk factors following coronary intervention.^2^^,^^3^ Additionally, SII has been identified as an independent risk factor for protein-energy wasting in patients undergoing maintenance hemodialysis.^4^ Beyond its accuracy as an inflammatory marker, SII is valuable in predicting the diagnosis and prognosis of a wide range of diseases. It stands out as a novel, simple, and cost-effective indicator of the balance between inflammation and immune response.

Metabolic Syndrome (MetS) is a pathological condition characterized by disturbances in the metabolism of proteins, fats, carbohydrates, and other substances, significantly increasing the risk for cardiovascular diseases, stroke, type 2 diabetes, and other complications.^5^, ^6^, ^7^ It involves a combination of conditions, including high blood pressure, elevated blood sugar, excess abdominal fat, and abnormal cholesterol or triglyceride levels.^8^ These coexisting factors significantly amplify the risk of heart disease and other serious health issues. Although obesity and physical inactivity are primary drivers of MetS, genetic factors also play a role in its development.

Recent research has investigated the relationship between Metabolic Syndrome (MetS) and systemic inflammation, with the Systemic Immune-Inflammatory Index (SII) emerging as a promising marker of this association.^9^ The SII reflects the balance between immune response and systemic inflammation. Given that MetS is characterized by chronic low-grade inflammation, SII may serve as a valuable biomarker for assessing the extent of systemic inflammation in individuals with MetS.^10^ Understanding this relationship could provide new insights into the pathophysiology of MetS and aid in identifying individuals at heightened risk for cardiovascular complications.

This study was designed to evaluate the relationship between the Systemic Immune-Inflammatory Index (SII) and the prevalence of Metabolic Syndrome (MetS) within the U.S. population. Gaining insights into this relationship could be instrumental in informing prevention and treatment approaches in clinical settings. To accomplish this, the authors performed analyses of data from the National Health and Nutrition Examination Survey (NHANES).

## Materials and methods

### Data source

The National Health and Nutrition Examination Survey (NHANES) is a set of cross-sectional, multistage probability samples that represent the U.S. civilian population, excluding those living in institutions. Since 1999, the survey has been conducted by the National Center for Health Statistics (NCHS) and follows a biennial cycle (https://wwwn.cdc.gov/nchs/nhanes/tutorials/module2.aspx). Data collection involves administering health-related questionnaires, conducting physical examinations, and performing laboratory tests on participants. The resulting data are made publicly accessible on the NCHS website (https://www.cdc.gov/nchs/nhanes/index.htm). Written informed consent was obtained from all NHANES participants, and the study protocols received approval from the Institutional Review Board of the NCHS.

### Study population

Metabolic Syndrome (MetS) was identified using the criteria set forth by the American Heart Association and the National Heart, Lung, and Blood Institute. The criteria include a waist circumference of ≥ 102 cm (≥ 40 inches) for men and ≥ 88 cm (≥ 35 inches) for women; triglyceride levels of ≥ 150 mg/dL (1.7 mmoL/L) or current use of medications aimed at lowering triglycerides; HDL-C levels below 40 mg/dL (1.03 mmoL/L) in men and below 50 mg/dL (1.3 mmoL/L) in women, or the use of medications intended to increase HDL-C; systolic blood pressure of ≥ 130 mmHg and/or diastolic blood pressure of ≥ 85 mmHg, or the use of antihypertensive drugs in individuals with a history of hypertension; and fasting blood glucose levels of ≥ 100 mg/dL or the use of medications to manage hyperglycemia.^5^ This study included participants aged 20 to 80-years from the 2013–2014, 2015–2016, and 2017–2018 NHANES cycles. Data were pooled from a total of 29,400 participants across five consecutive 2-year cycles to form this population-based cohort study, alongside the extraction of pertinent patient outcome data.

**Fig. 1:**
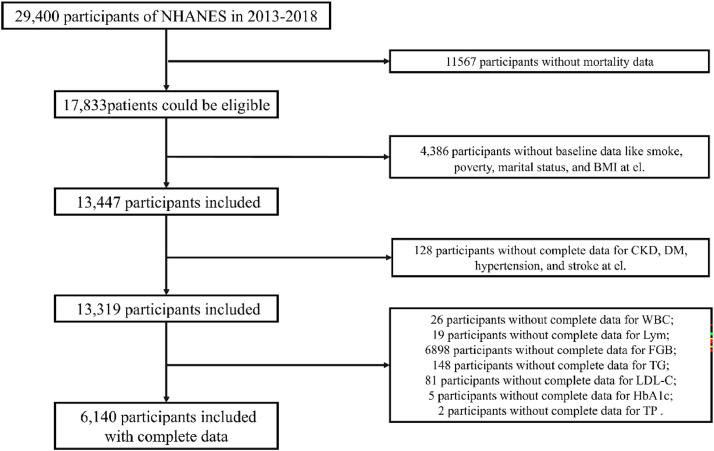
The flow chart.

For the present analysis, the authors incorporated sociodemographic data, individual health histories, examination results, and laboratory findings from three NHANES cycles (2013–2018), ensuring a representative sample. The exclusion criteria applied were: 1) Individuals under 20-years of age; 2) Pregnant participants; 3) Missing data regarding complications; 4) Absence of metabolic syndrome data; and 5) Incomplete laboratory test results. Participants with any of these exclusions were not included in the study. After applying these criteria, a total of 6140 participants remained in the analysis, of which 2295 were identified as having metabolic syndrome (Fig. 1). The study was conducted in accordance with the principles outlined in the Declaration of Helsinki.

### Systemic immune-inflammation index (SII)

The Systemic Immune-Inflammatory Index (SII) is a recognized marker indicative of systemic inflammation, which captures the interplay between immune response and inflammation. SII is determined by the formula SII = P × N/L, where P stands for peripheral platelet count, N denotes neutrophil count, and L indicates lymphocyte count. For this study, participants were categorized into four groups based on the quartiles of their SII levels: SIIQ1 (*n* = 1535) with values between 4.06 and 306.85, SIIQ2 (*n* = 1535) ranging from 306.85 to 427.94, SIIQ3 (*n* = 1536) with levels from 427.94 to 606.00, and SIIQ4 (*n* = 1534) with values spanning from 606.00 to 6171.20. Quartiles were selected to examine potential nonlinear relationships, particularly because no standardized reference range for SII exists, making the results easier to interpret.

### Outcome variables

The main outcome of interest in this study was all-cause mortality. To determine the survival status of participants, data from NHANES were connected to death records from the National Death Index (NDI). The causes of death were classified based on the International Classification of Diseases, Tenth Revision (ICD-10), as documented in the publicly available NHANES-linked mortality files. For each individual, the follow-up period started at the baseline NHANES interview and continued until the date of death or the last follow-up, with updates provided until December 31, 2019.^11^ Baseline data for participants were matched with survival outcomes using the respondent Sequence Number (SEQN) provided by NHANES. To categorize deaths in accordance with the International Classification of Diseases, Tenth Revision (ICD-10), the National Center for Health Statistics (NCHS) created the UCOD_113 variable. All-cause mortality was defined as death from any cause, including, but not limited to, heart disease (codes 54–68), cancer (malignant neoplasms; codes 19–43), chronic lower respiratory diseases (codes 82–86), unintentional injuries (codes 112–123), cerebrovascular diseases (code 70), Alzheimer's disease (code 52), diabetes (code 46), pneumonia and influenza (codes 76–78), as well as other causes. Cardiovascular mortality specifically refers to deaths caused by heart disease. For further details on the survival data, please refer to: NCHS Mortality Data Linkage.

### Covariates

The multivariate model used in this analysis included several covariates as adjustment factors: age, race/ethnicity (classified as White, Black, Mexican American, and other races), marital status (married, never married, and widowed/divorced/separated), poverty income ratio (PIR, categorized into three levels: 0–1, 1–3, and over 3), the Systemic Immune-Inflammation Index (SII), and variables related to medical history (including hypertension, diabetes, Cardiovascular Disease [CVD], Chronic Kidney Disease [CKD], stroke, and Chronic Obstructive Pulmonary Disease [COPD]). CKD progression risk was divided into four levels: low, moderate, high, and very high. For this research, dietary intake information was taken from the first day of dietary recall. Additional details on these covariates can be found on the official NHANES website. Sedentary time could not be reliably quantified; residual confounding by prolonged sitting is possible. Future cycles with accelerometer-derived sedentary behavior should verify the present findings.

### Statistical analysis

In this study, the authors utilized variables from four main sections of the NHANES dataset: demographic, examination, laboratory, and questionnaire data. The demographic section captured information such as sex, age, race/ethnicity, marital status, and the Poverty Income Ratio (PIR). The examination and laboratory sections primarily provided data on Body Mass Index (BMI), HbA1c levels, fasting blood glucose, fast blood Triglyceride (TG), Low-Density Lipoprotein Cholesterol (LDL-C), High-Density Lipoprotein Cholesterol (HDL-C), Hemoglobin (Hb), platelet count, Neutrophil (Neu) count, Lymphocyte (Lym) count, and Creatinine (Cre) levels. All routine biochemical measurements followed the procedures specified in the NHANES Laboratory/Medical Technologist Manual of Procedures. BMI was determined by dividing weight (kg) by height squared (m^2^). Smoking status was divided into never smokers, former smokers, and current smokers. Alcohol consumption was assessed based on whether participants had never consumed alcohol or had a history of alcohol use. Hypertension was defined by a self-reported history, the use of antihypertensive drugs, or an average Systolic Blood Pressure (SBP) of 140 mmHg or higher and/or an average Diastolic Blood Pressure (DBP) of 90 mmHg or higher. Diabetes mellitus was diagnosed using multiple criteria, including a self-reported physician diagnosis, Fasting Blood Glucose (FBG) levels of 7.0 mmoL/L or higher, HbA1c levels of 6.5% or greater, or the current use of diabetes medications. A history of stroke or cancer was determined through participant responses on questionnaires. To depict potential non-linearity, the authors fitted Restricted Cubic Splines (RCS) with the number and location of knots chosen a priori by minimizing Akaike’s information criterion; only splines retaining a significant likelihood-ratio test (*p* < 0.05) were kept. The resultant nadir is therefore data-driven and should be considered exploratory until replicated in independent cohorts.

## Results

### Baseline characteristics of the study population

A total of 29,400 participants were initially screened from the NHANES 2013‒2018 dataset. After excluding 11,567 participants due to lack of mortality data, 17,833 patients were considered potentially eligible. Subsequently, 4386 participants were excluded because of missing baseline data, including smoking, poverty, marital status, and BMI, resulting in 13,447 participants being included. Further exclusion of 128 participants without complete data for Chronic Kidney Disease (CKD), Diabetes Mellitus (DM), hypertension, and stroke left 13,319 participants. Finally, participants with incomplete data for specific indicators were excluded: 26 for White Blood Cell Count (WBC), 19 for Lymphocyte count (Lym), 148 for Hemoglobin A1c (HbA1c), 81 for Triglycerides (TG), 5 for Low-Density Lipoprotein Cholesterol (LDL-C), and 2 for Total Protein (TP). Ultimately, 6140 participants with complete data were included in the study (Fig. 1).

The analysis included a total of 6140 participants, with 2295 (37.52%) diagnosed with metabolic syndrome. The median age of the cohort was 48.4-years, with 51.20% being female. Participants with metabolic syndrome had an average age of 54.9-years, while those without the condition averaged 44.8-years. Notable differences were observed between the two groups in terms of Body Mass Index (BMI), the occurrence of hypertension, and the presence of comorbid conditions like diabetes. In laboratory tests, all biomarkers ‒ excluding hemoglobin levels, platelet count, total protein, and Low-Density Lipoprotein Cholesterol (LDL-C) ‒ were found to be higher in the metabolic syndrome group compared to the non-diagnosed group (Table 1).

**Table 1:** Baseline characteristics of the study population.

Variable	Overall (*n* = 6140)	MS-No (*n* = 3845)	MS-Yes (*n* = 2295)	p-value
Age (year)	48.40 (0.41)	44.82 (0.49)	54.90 (0.52)	**<0.0001**
Sex, n (%)				**0.800**
Female	3164 (51.20)	1944 (51.01)	1220 (51.53)	
Male	2976 (48.80)	1901 (48.99)	1075 (48.47)	
Race, n (%)				**0.001**
White	2398 (66.56)	1424 (65.13)	974 (69.14)	
Black	1237 (10.17)	810 (10.90)	427 (8.85)	
Mexican	867 (8.38)	508 (8.19)	359 (8.71)	
Other	1638 (14.89)	1103 (15.78)	535 (13.30)	
Marital status, n (%)				**<0.0001**
Married	3220 (55.87)	1925 (54.02)	1295 (59.21)	
Never married	1072 (16.86)	840 (20.67)	232 (9.96)	
Widowed, divorced, or separated	1848 (27.26)	1080 (25.30)	768 (30.82)	
PIR				**0.001**
0.00‒1.00	1303 (14.36)	806 (14.25)	497 (14.54)	
1.00‒3.00	2599 (36.19)	1532 (34.03)	1067 (40.11)	
>3.00	2238 (49.45)	1507 (51.71)	731 (45.34)	
Smoke, n (%)				**<0.0001**
Former	1512 (26.05)	817 (22.81)	695 (31.91)	
Never	3451 (55.67)	2279 (59.26)	1172 (49.17)	
Now	1177 (18.28)	749 (17.92)	428 (18.92)	
Alcohol consumption, n (%)				**<0.0001**
No	4232 (65.27)	2481 (60.70)	1751 (73.56)	
Yes	1908 (34.73)	1364 (39.30)	544 (26.44)	
BMI				**<0.0001**
<18.50	102 (1.62)	97 (2.39)	5 (0.22)	
18.50‒25.0	1667 (26.88)	1521 (38.85)	146 (5.19)	
≥25.0	4371 (71.51)	2227 (58.77)	2144 (94.59)	
Hypertension, n (%)				**<0.0001**
No	3556 (62.06)	2793 (76.42)	763 (36.04)	
Yes	2584 (37.94)	1052 (23.58)	1532 (63.96)	
DM, n (%)				**<0.0001**
No	4787 (83.20)	3483 (94.04)	1304 (63.56)	
Yes	1353 (16.80)	362 (5.96)	991 (36.44)	
Stroke, n (%)				**<0.0001**
No	5898 (97.06)	3748 (98.22)	2150 (94.96)	
Yes	242 (2.94)	97 (1.78)	145 (5.04)	
CKD, n (%)				**<0.0001**
No	5045 (85.55)	3379 (90.52)	1666 (76.56)	
Yes	1095 (14.45)	466 (9.48)	629 (23.44)	
CKD prognosis, n (%)				**<0.0001**
Low risk	5045 (85.55)	3379 (90.52)	1666 (76.56)	
Moderate risk	753 (10.66)	340 (7.45)	413 (16.49)	
High risk	203 (2.49)	77 (1.37)	126 (4.53)	
Very high risk	139 (1.29)	49 (0.66)	90 (2.42)	
CVD, n (%)				**<0.0001**
No	5426 (90.82)	3582 (94.73)	1844 (83.74)	
Yes	714 (9.18)	263 (5.27)	451 (16.26)	
COPD, n (%)				**0.001**
No	5905 (96.40)	3742 (97.60)	2163 (94.24)	
Yes	235 (3.60)	103 (2.40)	132 (5.76)	
Dead, n (%)				**0.004**
No	5857 (96.49)	3696 (97.35)	2161 (94.93)	
Yes	283 (3.51)	149 (2.65)	134 (5.07)	
Wbc (× 10^12^/L)	6.86 (0.05)	6.49 (0.06)	7.53 (0.06)	**<0.0001**
Lym (× 10^12^/L)	2.03 (0.02)	1.98 (0.02)	2.13 (0.03)	**<0.0001**
Neu (× 10^12^/L)	4.02 (0.04)	3.74 (0.04)	4.53 (0.04)	**<0.0001**
Hb (g/L)	14.30 (0.03)	14.26 (0.04)	14.36 (0.05)	0.03
Plt (× 10^12^/L)	235.36 (1.31)	233.87 (1.51)	238.08 (1.76)	0.04
FBG (mmoL/L)	6.00 (0.03)	5.52 (0.02)	6.88 (0.06)	**<0.0001**
HbA1c (%)	5.66 (0.02)	5.41 (0.01)	6.11 (0.04)	**<0.0001**
TP (g/L	7.07 (0.01)	7.07 (0.01)	7.06 (0.02)	0.970
TG (mmoL/L)	1.23 (0.01)	0.96 (0.01)	1.72 (0.03)	**<0.0001**
HDL-C (mmoL/L)	1.42 (0.01)	1.54 (0.01)	1.21 (0.01)	**<0.0001**
LDL-C (mmoL/L)	2.90 (0.02)	2.88 (0.02)	2.93 (0.03)	0.110
Cre (umoL/L)	77.72 (0.57)	76.52 (0.50)	79.91 (1.12)	**0.003**
SII	505.56 (5.35)	478.77 (5.93)	554.08 (8.17)	**<0.0001**

BMI, Body Mass Index; PIR, Poverty Income Ratio; DM, Diabetes Mellitus; MS, Metabolic Syndrome; FBG, Fasting Blood Glucose; HDL-C, High-Density Lipoprotein Cholesterol; LDL-C, Low-Density Lipoprotein Cholesterol; TG, Triglycerides; WBC, White Blood Cell Count; Lym, Lymphocyte; Neu, Neutrophil; Hb, Hemoglobin. Plt, Platelet; TP, Total Protein; SII, Systemic Immune Inflammation Index.

Participants were divided into four quartile groups (Q1-Q4) based on their SII values. Those in the higher SII quartiles were generally older, more frequently female, and more likely to be current smokers with a higher Body Mass Index (BMI) than those in the lowest quartile. The highest SII quartile also had a greater prevalence of comorbid conditions, including hypertension, diabetes, stroke, chronic renal insufficiency, and chronic obstructive pulmonary disease. Additionally, significant differences were observed in laboratory results among the quartiles, with individuals in the higher SII quartiles showing increased levels of White Blood Cells (WBC), Lymphocytes (Lym), Neutrophils (Neu), Hemoglobin (Hb), Platelets (Plt), Fasting Blood Glucose (FBG), HbA1c, Triglyceride (TG), High-Density Lipoprotein Cholesterol (HDL-C), and Creatinine (Cre) compared to those in the lowest quartile (*p* < 0.05, Table 2).

**Table 2:** The results of SII different level subgroups analyses.

Variable	Total	Q1	Q2	Q3	Q4	p-value
Age (year)	48.40 (0.41)	46.82 (0.71)	46.87 (0.58)	48.79 (0.68)	50.80 (0.62)	**<0.001**
Sex, n (%)						**<0.0001**
Female	3164 (51.20)	695 (42.79)	745 (47.54)	837 (53.66)	887 (59.29)	
Male	2976 (48.80)	840 (57.21)	790 (52.46)	699 (46.34)	647 (40.71)	
Race, n (%)						**<0.0001**
White	2398 (66.56)	442 (57.72)	573 (66.45)	636 (68.07)	747 (72.54)	
Black	1237 (10.17)	473 (17.27)	284 (8.78)	252 (8.52)	228 (7.18)	
Mexican	867 (8.38)	203 (8.40)	240 (9.42)	222 (8.31)	202 (7.42)	
Other	1638 (14.89)	417 (16.61)	438 (15.34)	426 (15.09)	357 (12.86)	
Marital status, n (%)						0.060
Married	3220 (55.87)	813 (54.81)	839 (56.46)	806 (57.11)	762 (55.01)	
Never married	1072 (16.86)	297 (20.35)	255 (17.52)	267 (15.34)	253 (14.80)	
Widowed, divorced, or separated	1848 (27.26)	425 (24.84)	441 (26.01)	463 (27.55)	519 (30.20)	
PIR						0.260
0.00‒1.00	1303 (14.36)	314 (13.67)	337 (14.23)	321 (13.63)	331 (15.73)	
1.00‒3.00	2599 (36.19)	651 (37.57)	626 (35.16)	635 (34.20)	687 (37.94)	
>3.00	2238 (49.45)	570 (48.76)	572 (50.61)	580 (52.16)	516 (46.34)	
Smoke, n (%)						**<0.0001**
Former	1512 (26.05)	367 (26.39)	370 (24.74)	368 (26.63)	407 (26.45)	
Never	3451 (55.67)	912 (57.81)	904 (60.34)	876 (56.41)	759 (48.76)	
Now	1177 (18.28)	256 (15.80)	261 (14.91)	292 (16.96)	368 (24.78)	
Alcohol consumption, n (%)						**0.003**
No	4232 (65.27)	1035 (60.10)	1053 (64.35)	1065 (67.00)	1079 (68.80)	
Yes	1908 (34.73)	500 (39.90)	482 (35.65)	471 (33.00)	455 (31.20)	
BMI						**<0.001**
<18.50	102 (1.62)	25 (1.33)	26 (1.36)	27 (1.54)	24 (2.16)	
18.50‒25.0	1667 (26.88)	457 (31.52)	456 (29.60)	397 (23.95)	357 (23.23)	
≥25.0	4371 (71.51)	1053 (67.15)	1053 (69.04)	1112 (74.51)	1153 (74.61)	
Hypertension, n (%)						**<0.001**
No	3556 (62.06)	942 (65.62)	922 (64.80)	893 (62.74)	799 (55.84)	
Yes	2584 (37.94)	593 (34.38)	613 (35.20)	643 (37.26)	735 (44.16)	
DM, n (%)						**<0.0001**
No	4787 (83.20)	1250 (88.33)	1219 (85.45)	1197 (82.29)	1121 (77.68)	
Yes	1353 (16.80)	285 (11.67)	316 (14.55)	339 (17.71)	413 (22.32)	
Stroke, n (%)						0.230
No	5898 (97.06)	1492 (97.69)	1477 (97.42)	1475 (97.07)	1454 (96.19)	
Yes	242 (2.94)	43 (2.31)	58 (2.58)	61 (2.93)	80 (3.81)	
CKD, n (%)						**<0.0001**
No	5045 (85.55)	1323 (89.97)	1306 (87.07)	1255 (85.50)	1161 (80.51)	
Yes	1095 (14.45)	212 (10.03)	229 (12.93)	281 (14.50)	373 (19.49)	
CKD prognosis, n (%)						**<0.0001**
Low risk	5045 (85.55)	1323 (89.97)	1306 (87.07)	1255 (85.50)	1161 (80.51)	
Moderate risk	753 (10.66)	155 (7.79)	171 (10.53)	191 (10.71)	236 (13.14)	
High risk	203 (2.49)	37 (1.46)	35 (1.58)	58 (2.79)	73 (3.94)	
Very high risk	139 (1.29)	20 (0.78)	23 (0.83)	32 (1.01)	64 (2.41)	
CVD, n (%)						**0.010**
No	5426 (90.82)	1385 (92.09)	1369 (92.20)	1371 (91.21)	1301 (88.08)	
Yes	714 (9.18)	150 (7.91)	166 (7.80)	165 (8.79)	233 (11.92)	
COPD, n (%)						**<0.0001**
No	5905 (96.40)	1498 (97.42)	1498 (97.70)	1487 (97.58)	1422 (93.21)	
Yes	235 (3.60)	37 (2.58)	37 (2.30)	49 (2.42)	112 (6.79)	
MS						**<0.0001**
No	3845 (64.43)	1064 (73.02)	988 (67.86)	954 (63.86)	839 (54.60)	
Yes	2295 (35.57)	471 (26.98)	547 (32.14)	582 (36.14)	695 (45.40)	
Dead, n (%)						**<0.0001**
No	5857 (96.49)	1476 (96.46)	1485 (97.90)	1474 (97.44)	1422 (94.27)	
Yes	283 (3.51)	59 (3.54)	50 (2.10)	62 (2.56)	112 (5.73)	
WBC (× 10^12^/L)	6.86 (0.05)	5.76 (0.08)	6.36 (0.07)	6.86 (0.08)	8.25 (0.09)	**<0.0001**
Lym (× 10^12^/L)	2.03 (0.02)	2.32 (0.05)	2.10 (0.03)	1.96 (0.02)	1.80 (0.02)	**<0.0001**
Neu (× 10^12^/L)	4.02 (0.04)	2.70 (0.04)	3.49 (0.04)	4.09 (0.05)	5.56 (0.07)	**<0.0001**
Hb (g/L)	14.30 (0.03)	14.38 (0.06)	14.45 (0.05)	14.24 (0.06)	14.14 (0.04)	**<0.0001**
PLT (× 10^12^/L)	235.36 (1.31)	197.24 (1.78)	223.02 (1.76)	243.32 (1.81)	271.14 (2.61)	**<0.0001**
FBG (mmoL/L)	6.00 (0.03)	5.79 (0.04)	5.97 (0.06)	6.08 (0.07)	6.15 (0.05)	**<0.0001**
HbA1c (%)	5.66 (0.02)	5.56 (0.03)	5.63 (0.03)	5.68 (0.03)	5.75 (0.03)	**<0.001**
TP (g/dL)	7.07 (0.01)	7.09 (0.02)	7.07 (0.02)	7.05 (0.02)	7.05 (0.02)	0.380
TG (mmoL/L)	1.23 (0.01)	1.12 (0.02)	1.25 (0.03)	1.27 (0.03)	1.28 (0.03)	**<0.001**
HDL-C (mmoL/L)	1.42 (0.01)	1.47 (0.02)	1.43 (0.02)	1.40 (0.02)	1.40 (0.02)	**0.020**
LDL-C (mmoL/L)	2.90 (0.02)	2.88 (0.03)	2.94 (0.04)	2.92 (0.03)	2.86 (0.03)	0.190
Cre (μmoL/L)	77.72 (0.57)	79.30 (0.69)	77.94 (0.84)	75.44 (0.65)	78.39 (1.45)	**<0.001**
SII	505.56 (5.35)	234.50 (1.99)	366.58 (1.32)	505.88 (2.03)	862.21 (10.04)	**<0.0001**

### Results of univariate and multivariate analyses of populations with metabolic syndrome

**Fig. 2:**
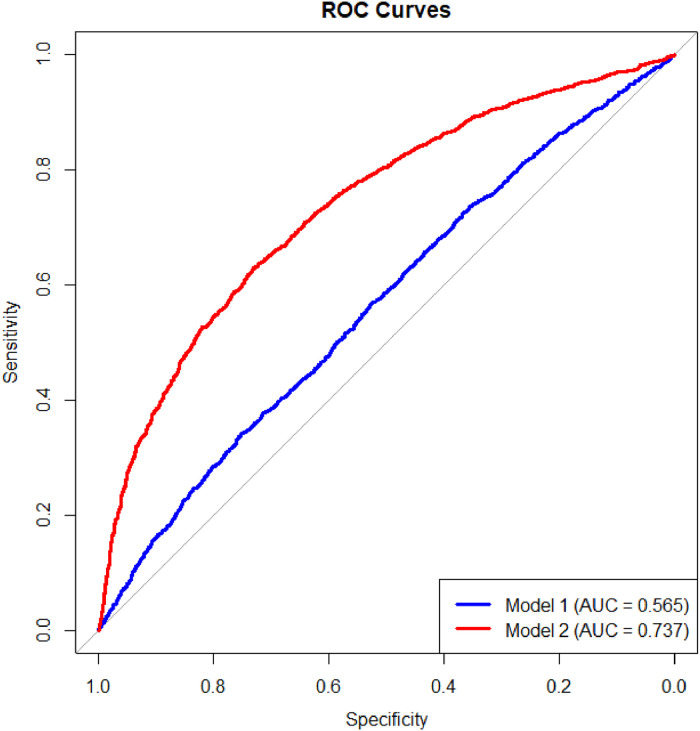
Comparison of ROC curves of two models based on SII.

The authors performed a univariate analysis to assess the impact of various factors on the metabolic syndrome. The analysis revealed that age, smoking status, alcohol consumption, Body Mass Index (BMI), and comorbidities such as hypertension, diabetes mellitus, chronic renal insufficiency, and stroke significantly influenced the prevalence of metabolic syndrome (*p* < 0.05). Additionally, significant differences were observed across all laboratory assays, with the exception of total protein content (*p* < 0.05, Table 3). Subsequent multifactorial logistic regression analysis indicated that age and underlying comorbidities, including hypertension, diabetes mellitus, and stroke, were associated with an increased risk of developing metabolic syndrome. Significant disparities were also identified between the two groups in laboratory tests, including Hemoglobin (Hb), Lymphocytes (Lym), Fasting Blood Glucose (FBG), HbA1c, Total Cholesterol (TC), and High-Density Lipoprotein Cholesterol (HDL-C). Notably, the trends for Hb and HDL-C were opposite to those observed for other indicators (Table 4). When excluding direct indicators of MetS, the Area Under the Curve (AUC) for the Systemic Immune-Inflammatory Index (SII) alone in predicting MetS was 0.565 in model 1. In contrast, model 2, which adjusted for age, hemoglobin, lymphocyte count, and HbA1c level, demonstrated an increased AUC of 0.737 (Fig. 2).

**Table 3:** Results of univariate analysis of study patients.

Character	Estimate	Std. Error	t value	OR	95% CI	p
Age	0.04	0	13.9	1.04	1.04 (1.03, 1.04)	**<0.0001**
Sex	−0.02	0.08	−0.25	0.98	0.98 (0.83, 1.16)	0.800
Race						
White	Ref	Ref	Ref	Ref	Ref	Ref
Black	−0.27	0.08	−3.46	0.77	0.77 (0.65, 0.89)	**0.001**
Mexican	0	0.09	0.01	1	1.00 (0.84, 1.19)	0.990
Other	−0.23	0.08	−2.85	0.79	0.79 (0.67, 0.93)	**0.010**
Marital status						
Married	Ref	Ref	Ref	Ref	Ref	Ref
Never married	−0.82	0.13	−6.46	0.44	0.44 (0.34, 0.57)	**<0.0001**
Widowed, divorced, or separated	0.11	0.09	1.22	1.11	1.11 (0.93, 1.32)	0.23
PIR						
0.00‒1.00	Ref	Ref	Ref	Ref	Ref	Ref
1.00‒3.00	0.14	0.1	1.5	1.16	1.16 (0.95, 1.40)	0.140
>3.00	−0.15	0.12	−1.3	0.86	0.86 (0.68, 1.09)	0.200
Smoke						
Former	Ref	Ref	Ref	Ref	Ref	Ref
Never	−0.52	0.08	−6.29	0.59	0.59 (0.50, 0.70)	**<0.0001**
Now	−0.28	0.12	−2.41	0.75	0.75 (0.60, 0.96)	**0.02**
Alcohol consumption	−0.59	0.1	−6.06	0.56	0.56 (0.46, 0.68)	**<0.0001**
BMI						
<18.50	Ref	Ref	Ref	Ref	Ref	Ref
18.50‒25.0	0.37	0.55	0.67	1.44	1.44 (0.48, 4.35)	0.510
≥25.0	2.85	0.55	5.23	17.36	17.36 (5.77, 52.19)	**<0.0001**
Hypertension	1.75	0.09	18.56	5.75	5.75 (4.76, 6.95)	**<0.0001**
DM	2.2	0.09	24.29	9.05	9.05 (7.54, 10.86)	**<0.0001**
Stroke	1.08	0.15	7.12	2.94	2.94 (2.16, 3.98)	**<0.0001**
CKD	1.07	0.11	10.03	2.92	2.92 (2.36, 3.63)	**<0.0001**
CKD_prognosis						
Low risk	Ref	Ref	Ref	Ref	Ref	Ref
Moderate risk	0.96	0.12	7.88	2.62	2.62 (2.05, 3.35)	**<0.0001**
High risk	1.36	0.17	7.8	3.91	3.91 (2.75, 5.56)	**<0.0001**
Very high risk	1.46	0.2	7.33	4.33	4.33 (2.89, 6.47)	**<0.0001**
CVD	1.25	0.11	11.26	3.49	3.49 (2.79, 4.36)	**<0.0001**
COPD	0.91	0.27	3.36	2.48	2.48 (1.44, 4.29)	**0.002**
Wbc (× 10^12^/L)	0.26	0.03	10.01	1.29	1.29 (1.23, 1.36)	**<0.0001**
Lym (× 10^12^/L)	0.27	0.1	2.7	1.3	1.30 (1.07, 1.59)	**0.010**
Neu (× 10^12^/L)	0.32	0.03	11.58	1.38	1.38 (1.30, 1.45)	**<0.0001**
Hb (g/L)	0.05	0.02	2.19	1.05	1.05 (1.00, 1.10)	**0.030**
Plt (× 10^12^/L)	0	0	2.16	1	1.00 (1.00,1.00)	**0.040**
FBG (mmoL/L)	1.03	0.08	13.23	2.81	2.81 (2.40, 3.29)	**<0.0001**
HbA1c (%)	1.27	0.09	14.29	3.56	3.56 (2.98, 4.26)	**<0.0001**
TP (g/dL)	0	0.09	−0.04	1	1.00 (0.83, 1.19)	0.970
TG (mmoL/L)	1.8	0.09	20.83	6.02	6.02 (5.06, 7.16)	**<0.0001**
HDL-C (mmoL/L)	−2.54	0.18	−13.79	0.08	0.08 (0.05, 0.11)	**<0.0001**
LDL-C (mmoL/L)	0.06	0.04	1.61	1.06	1.06 (0.99, 1.14)	0.110
Cre (μmoL.L)	0	0	7.65	1	1.00 (1.00, 1.00)	**<0.0001**

**Table 4:** Results of multifactorial analysis of study participants.

Character	Estimate	Std. Error	*t*-value	OR	95% CI	p
Age	0.03	0	6.98	1.03	1.03 (1.02, 1.04)	**<0.0001**
Smoke						
Former	Ref	Ref	Ref	Ref	Ref	Ref
Never	−0.09	0.11	−0.74	0.92	0.92 (0.72, 1.16)	0.460
Now	−0.26	0.17	−1.49	0.77	0.77 (0.54, 1.11)	0.150
Alcohol consumption	−0.34	0.13	−2.54	0.71	0.71 (0.54, 0.94)	**0.020**
Hypertension	1.45	0.12	11.8	4.25	4.25 (3.30, 5.48)	**<0.0001**
DM	0.55	0.19	2.92	1.73	1.73 (1.17, 2.56)	**0.010**
Stroke	0.34	0.29	1.19	1.41	1.41 (0.78, 2.55)	0.240
CKD	−0.65	0.22	−2.93	0.52	0.52 (0.33, 0.83)	**0.010**
CKD prognosis						
Low risk	Ref	Ref	Ref	Ref	Ref	Ref
Moderate risk	0.97	0.29	3.34	2.64	2.64 (1.45, 4.82)	**0.003**
High risk	0.36	0.3	1.2	1.43	1.43 (0.77, 2.64)	0.240
CVD	0.15	0.2	0.73	1.16	1.16 (0.77, 1.74)	0.470
COPD	0.4	0.38	1.07	1.49	1.49 (0.69, 3.25)	0.300
WBC	−0.05	0.17	−0.3	0.95	0.95 (0.67, 1.35)	0.770
Neu	0.3	0.21	1.47	1.35	1.35 (0.88, 2.07)	0.160
Hb	−0.16	0.03	−4.7	0.85	0.85 (0.80, 0.92)	**<0.0001**
PLT	0	0	2.61	1	1.00 (1.00, 1.00)	**0.020**
Lym	0.05	0.17	0.27	1.05	1.05 (0.73, 1.50)	0.790
FBG	0.66	0.13	5.24	1.94	1.94 (1.49, 2.52)	**<0.0001**
HbA1c	−0.37	0.12	−3.13	0.69	0.69 (0.54, 0.88)	**0.005**
TG	1.36	0.09	14.53	3.89	3.89 (3.20, 4.72)	**<0.0001**
HDL-C	−1.95	0.29	−6.73	0.14	0.14 (0.08, 0.26)	**<0.0001**
SII	0	0	−1.98	1	1.00 (1.00, 1.00)	0.060

### Analysis results of SII index and all-cause and cardiovascular disease mortality risk in metabolic syndrome population

**Fig. 3:**
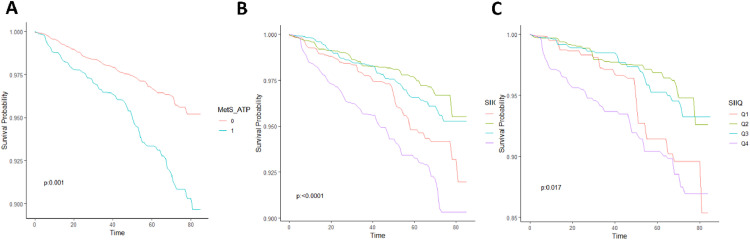
Association of SII with all-cause mortality and cardiovascular mortality risk in the population.

During the follow-up period, a total of 283 all-cause deaths were recorded, including 73 cardiovascular deaths, 5 deaths due to diabetes mellitus, 1 death from pneumonia, 12 cerebrovascular deaths, 75 cancer deaths, and 111 deaths from other causes. The mortality rate was significantly higher in patients with metabolic syndrome (5.07%) compared to the non-affected population (2.65%) (Fig. 3A). For all participants, individuals in SIIQ2 and SIIQ3 had a lower risk of death than those in SIIQ1 and SIIQ4, indicating that higher or lower SII scores were associated with a reduced risk of death (Fig. 3B). The same trend was observed in the population with a diagnosis of metabolic syndrome as in the overall population (Fig. 3C).

In the stratified analysis based on different SII levels, differences in age, alcohol consumption, hypertension, diabetes, and chronic kidney injury were observed between the two groups across various SII categories. Interestingly, within the younger age group, individuals with comorbid conditions such as hypertension, diabetes mellitus, and chronic kidney disease demonstrated a higher risk of developing metabolic syndrome at comparable SII levels (Table 5).

**Table 5:** All-cause mortality risk by septile of SII levels.

All-cause SII septile	Crude model (95%CI)	Model 1 (95%CI)	Model 2 (95%CI)
Q1	Ref	Ref	Ref
Q2	1.15 (0.99,1.32)	1.12 (0.95,1.31)	1.06 (0.90,1.24)
Q3	1.30 (1.14,1.49) ^⁎⁎⁎^	1.25 (1.08,1.43) ^⁎⁎^	1.18 (1.01,1.38)*
Q4	1.58 (1.35,1.85) ^⁎⁎⁎⁎^	1.44 (1.22,1.69) ^⁎⁎⁎⁎^	1.31 (1.11,1.55)^⁎⁎^
p for trend	**<0.0001**	**<0.0001**	**<0.001**
**Cardiovascular SII septile**	**Crude model (95%CI)**	**Model 1 (95%CI)**	**Model 2 (95%CI)**
**Q1**	Ref	Ref	Ref
**Q2**	1.18 (1.02,1.36) *	1.14 (0.98,1.33)	1.07 (0.92,1.26)
**Q3**	1.33 (1.16,1.52) ^⁎⁎⁎⁎^	1.27 (1.11,1.46)^⁎⁎⁎^	1.20 (1.03,1.40)*
**Q4**	1.58 (1.35,1.85)^⁎⁎⁎⁎^	1.44 (1.22,1.69)^⁎⁎⁎⁎^	1.30 (1.09,1.55)^⁎⁎^
***p* for trend**	**<0.0001**	**<0.0001**	**0.003**

Crudel model: SIIQ.

Model 1: SIIQ, Age.

Model 2: SIIQ, Age, Alcohol consumption, Hypertension, DM, CKD.

*p<0.05;** p<0.01; *** p<0.001;**** p<0.0001.

The authors constructed two Cox regression models to examine the association between SII index level and all-cause and cardiovascular mortality in people diagnosed with metabolic syndrome. The first model was an unadjusted crude model, while model 1 included age adjustment. Model 2 further adjusted for other factors, including age, alcohol consumption, hypertension, diabetes, and chronic kidney disease. Cox regression analysis of the risk of all-cause mortality in the metabolic syndrome population showed that individuals in the highest Quartile (Q4) had an increased risk of all-cause mortality compared with those in the lowest Quartile (Q1) in both models 1 and 2. Specifically, in model 1, the Hazard Ratio (HR) was 1.58 (95% CI 1.22‒1.69, *p* < 0.0001; trend *p* < 0.0001), and in model 2, the HR was 1.31 (95% CI 1.11‒1.55, *p* < 0.01; trend *p* < 0.001) (Table 5, see all-cause SII septile). Similarly, Cox regression analysis of cardiovascular mortality risk in the metabolic syndrome population showed that individuals in the highest Quartile (Q4) had a higher risk of cardiovascular mortality than those in the lowest Quartile (Q1) in both models 1 and 2. In model 1, the Hazard Ratio (HR) was 1.58 (95% CI 1.38–1.85, *p* < 0.0001; trend *p* < 0.0001), while in model 2, the HR was 1.44 (95% CI 1.22–1.69, *p* < 0.01; trend *p* < 0.001) (Table 5, see cardiovascular SII septile).

### Restricted spline cubic analysis of the relationship between SII index and mortality risk of metabolic syndrome

**Fig. 4:**
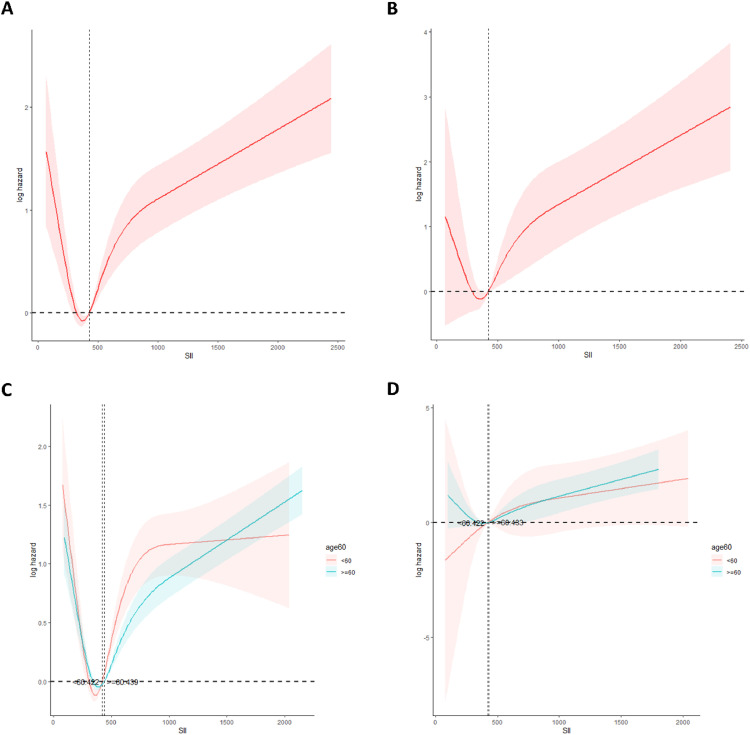
The RCS analysis of the relationship between SII and all-cause mortality and cardiovascular mortality risk in all people.

The authors assessed the dose-response relationship between SII index levels and mortality from all causes and cardiovascular events using Restricted Cubic Splines (RCS) (All causes: Fig. 4A; Cardiovascular events: Fig. 4B). Both age groups ‒ those younger than 60-years and those 60-years or older ‒ demonstrated a “J”-shaped nonlinear association between SII index levels and all-cause mortality (Fig. 4C). However, this “J”-shaped nonlinear pattern was observed for cardiovascular mortality only in individuals aged 60-years or older (Fig. 4D).

**Fig. 5:**
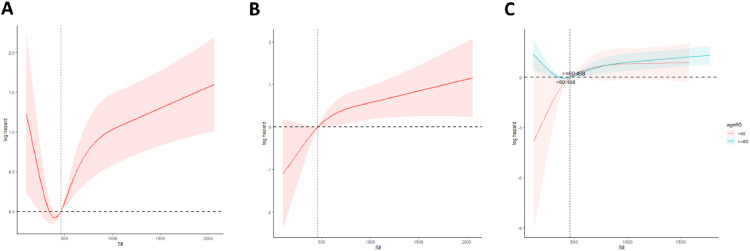
The RCS analysis of the relationship between SII and all-cause mortality and cardiovascular mortality risk in MetS people.

Similarly, in patients with metabolic syndrome, the dose-response relationship between SII index level and all-cause mortality analyzed with RCS showed a J-shaped nonlinear trend, consistent with the observed overall trend (*p* < 0.05). For all-cause mortality in this population, the threshold SII score was determined to be 457.6. When the SII index exceeded this threshold, the higher the SII score, the higher the risk of all-cause mortality. The “J”-shaped relationship with SII score previously observed in the metabolic syndrome population was no longer significant for cardiovascular disease mortality (nonlinear *p* = 0.201, see Fig. 5). This J-shaped pattern was significant only in the subgroup of individuals aged 60-years or older (*p* < 0.05, see Fig. 5C).

## Discussion

This longitudinal cohort study, utilizing NHANES 2013–2018 and NRI data, revealed a significant association between the Systemic Immune-Inflammatory Index (SII) and all-cause mortality among individuals with metabolic syndrome, as well as cardiovascular mortality in those aged 60-years and older.

The SII ‒ platelets × neutrophils/lymphocytes ‒ captures systemic inflammation in one number and has emerged as a promising predictor of outcomes in Percutaneous Coronary Intervention (PCI) and cancer cohorts.^12^ Patients with higher SII values are at significantly increased risk of Major Adverse Cardiovascular Events (MACEs), including heart failure, myocardial infarction, and all-cause mortality, highlighting its potential as a useful tool for assessing prognosis in post-PCI patients.^2^^,^^3^^,^^13^ In oncology, SII has been identified as a significant prognostic marker, particularly in prostate cancer.^14^ Elevated preoperative SII levels have been associated with poorer overall survival and progression-free survival, making it a valuable indicator for determining prognosis and guiding treatment strategies in cancer patients.^15^ Moreover, SII is easy to calculate, enhancing its utility in clinical settings.

Metabolic syndrome is a collection of metabolic abnormalities that significantly increases the risk of developing cardiovascular diseases and type 2 diabetes. This syndrome typically encompasses central obesity, insulin resistance, hypertension, and dyslipidemia, particularly elevated triglycerides and low levels of HDL cholesterol.^5^^,^^16^^,^^17^ The prevalence of metabolic syndrome is rising globally, largely due to increasing obesity rates and sedentary lifestyles. The concept of metabolic syndrome has evolved over time, with early research primarily linking it to insulin resistance.^18^ However, more recent studies suggest that the pathophysiology of the syndrome is complex and involves multiple factors, including genetic predispositions, chronic inflammation, and dysfunction of adipose tissue.^16^^,^^18^ Importantly, the presence of metabolic syndrome is associated with a higher risk of cardiovascular events that exceeds what might be expected from the individual components alone, although this association remains a topic of debate within the scientific community.^8^ Recent studies have explored the association between the Systemic Immune-Inflammatory Index (SII) score and the risk of obesity, chronic kidney disease, and early inflammatory diseases, primarily focusing on hospitalized patients with conditions such as pneumonia, early pregnancy miscarriage, systemic lupus erythematosus, and predicting restenosis after surgical stenting.^19^, ^20^, ^21^, ^22^, ^23^, ^24^, ^25^ However, there is a lack of research examining the association between SII and all-cause or cardiovascular mortality, particularly in patients with metabolic syndrome.

The authors mapped how age, sex, smoking and routine biomarkers cluster with MetS and found the expected links. The twist: low SII predicted higher death risk in the general cohort, but higher SII spelled danger in MetS, hinting that metabolic status reshapes the meaning of inflammation ‒ too little blunts host defence, too much fuels vascular injury, while the middle ground may be safest.^26^ Conversely, elevated SII levels are associated with excessive systemic inflammation, which is a well-established risk factor for diseases like cardiovascular diseases, diabetes, and certain cancers.^27^ Chronic inflammation leads to endothelial dysfunction, insulin resistance, and atherosclerosis, contributing to higher mortality.^28^^,^^29^ Thus, very high SII levels may reflect an excessive inflammatory state, worsening prognosis. Moderate SII levels might indicate an optimal balance between immune activation and controlled inflammation, explaining why these levels are associated with the lowest mortality risk.

The dual role of inflammation, being both protective and detrimental depending on its intensity and duration, has been described in the “inflammation paradox”.^30^ Low-grade inflammation, often seen in metabolic syndrome, may not be sufficient to protect against infections or chronic diseases, while excessive inflammation, as in autoimmune diseases or sepsis, can overwhelm the body and cause damage. The present findings mirror a J-shaped relationship that has also been described for other inflammatory biomarkers. In community cohorts, C-reactive protein,^31^ the neutrophil-to-lymphocyte ratio, and total white blood cell count display similarly elevated mortality at the lowest as well as the highest concentrations.^31^, ^32^, ^33^, ^34^ A low SII (i.e., combined lymphocyte, neutrophil and platelet count below the population median) may indicate reduced immune reserve, thereby increasing susceptibility to infection, cancer progression or frailty-related death.^35^^,^^36^ Conversely, extremely high SII values reflect hyper-inflammation that predisposes to cardiovascular events and metabolic decompensation. Thus, the “J”-shaped association is biologically coherent with the “inflammation paradox”, although the authors acknowledge that this interpretation remains speculative and requires validation in mechanistic studies. Experimental evidence supports a mechanistic basis for the observed J-shaped relationship. Platelets, neutrophils and lymphocytes ‒ the three components of SII ‒ jointly modulate endothelial function and insulin signaling. Platelet-derived IL-1β and CD40L reduce nitric-oxide availability and up-regulate adhesion molecules in human arteries, while neutrophil extracellular traps impair IRS-1 phosphorylation and AKT signalling in muscle and adipose tissue.^37^^,^^38^ Conversely, the lymphocyte fraction of SII preserves insulin sensitivity in animal models. Thus, low SII may indicate inadequate immune surveillance, whereas high SII reflects thrombo-inflammatory overload; both extremes converge on endothelial injury and insulin resistance, whereas moderate SII maintains an optimal immuno-thrombotic balance compatible with the lowest mortality risk.

The multifactorial analysis underscores the importance of considering various factors when assessing the risk of metabolic syndrome. The identification of laboratory tests as significant predictors opens potential avenues for early detection and intervention. The nonlinear relationship between SII scores and mortality, particularly the “J”-shaped curve, suggests the existence of a threshold effect: moderate levels of inflammation may be protective, while higher levels could prove detrimental. This finding has important implications for managing inflammatory conditions and the timing of interventions. However, the study's findings should be interpreted within the context of its limitations, including the potential for residual confounding factors and the necessity for further research to confirm the identified threshold for SII scores in relation to mortality.

While the authors utilized rigorous statistical adjustments and sensitivity analyses, the observational nature of NHANES data limits causal inference between SII and MetS outcomes. Residual confounding from unmeasured factors (e.g., genetic predispositions, subclinical infections, or microenvironmental inflammation) may persist. Additionally, the single-timepoint measurement of SII cannot capture dynamic changes in inflammatory status over the disease course. Nevertheless, these findings are consistent with the experimental evidence of a significant correlation between laboratory assay composite SII values and the incidence of MetS and associated cardiovascular time, which suggests the biological plausibility of SII as a modifiable risk factor rather than a passive marker.

Although the authors adjusted for multiple variables, residual confounding factors, which may not have been measured or adequately controlled for, could still influence the observed associations. Additionally, selection bias is possible, as the sample may not fully represent the broader population. Missing data, laboratory measurement errors, or inaccuracies in health indicators may also introduce bias or reduce the precision of our estimates. Furthermore, the long duration of data collection may have led to temporal biases, as participants' health statuses and environmental factors could have changed over time. While the present findings offer valuable insights, their generalizability to other populations or in different geographical and cultural contexts, may be limited, particularly due to the specific characteristics of the study population (e.g., age, race, gender distribution, and prevalence of metabolic syndrome), which may not apply to all demographic groups.

However, as this study is based on observational data, both cross-sectional and cohort designs, the authors must emphasize that causal inferences between SII and MetS or related mortality cannot be definitively drawn. While the authors identified robust dose-response relationships, including the J-shaped curve, and made adjustments for key confounders through multivariable models, the limitations inherent in observational research prevent us from concluding whether elevated SII directly contributes to the pathogenesis of MetS or whether it merely acts as a marker of an underlying inflammatory state already associated with metabolic dysregulation. These findings highlight the need for caution when interpreting the results in terms of causality. Therefore, it remains uncertain whether increased SII is an etiological factor for MetS or simply reflects a secondary response to established metabolic abnormalities. These analyses relied on the AHA/NHLBI waist-circumference thresholds derived predominantly from Europid populations. These cut-offs may underestimate central adiposity-related risk in South and Southeast Asians. Owing to the small proportion of self-reported South Asian participants in NHANES 2013–2018 (< 2%), the authors could not apply ethnic-specific thresholds while preserving nationally representative weights. Future multi-ethnic cohorts with adequate South Asian representation should validate whether lower waist-circumference cut-points (≥ 90 cm for men, ≥ 80 cm for women) strengthen the SII-MetS-mortality association.

Since the SII incorporates multiple inflammatory indices, anti-inflammatory medications may affect it. However, large cross-sectional studies face significant challenges in capturing the exact type, dosage, and duration of such medications ‒ relying mostly on self-reported data or broad medication categories, which introduces substantial variability. Consequently, the confounding effects of these drugs are difficult to accurately quantify, and more prospective, detailed data are needed to establish their direct relationship with SII and metabolic syndrome. Additionally, while nutritional status influences inflammatory levels and metabolic syndrome risk, the present study lacked systematic collection of nutritional data, only including BMI as a surrogate indicator of overall nutritional status ‒ though BMI is increasingly questioned for its comprehensiveness. Accurately capturing and adjusting for all aspects of nutritional status in large cohort studies is highly challenging, and refined dietary assessments (unfeasible in this research) are required to address this limitation.

Results highlight the complexity of metabolic syndrome and emphasize the need for a holistic approach to its understanding and management. Despite limitations, the study offers valuable insights into factors linked to metabolic syndrome and its mortality association. Future research should address these limitations via randomized controlled trials and diverse populations. Exploring the mechanisms of these associations is crucial, and studying personalized strategies based on individual risk factors and SII levels may further improve their management.

## Ethics statement

Approval for studies involving human subjects was obtained from the research ethics review board of the NCHS. The research adhered to applicable local laws and institutional guidelines. Participants gave their written informed consent to participate in the study.

## Consent to publication

The authors declared no potential conflicts of interest with respect to the research, authorship, and/or publication of this article.

## Authors’ contributions

Research concept and study design: YS and YSG. Data acquisition: SY and XZL. Data analysis/interpretation: SY. Statistical analysis: SY. Supervision and financial support: HYZ and YZA. SY had full access to all study data and take responsibility for data integrity and data analysis accuracy. All authors contributed to interpretation of data, article drafting, or revising of the intellectual content, and final approval of the version to be submitted.

## Fundings

The research received the grant from the 10.13039/501100001809National Natural Science Foundation of China (NSFC) (n° 82202366), Wu Jieping Medical Foundation Runze Fund for Critical Care Medicine (n° 320.6750.2022-2-34), Beijing Natural Science Foundation (L244067) and Beijing Key Clinical Specialty Outstanding Project.

## Data availability statement

This study analyzed publicly available datasets, which can be accessed at: https://wwwn.cdc.gov/nchs/nhanes/Default.aspx.

## Declaration of competing interest

The authors declare that the research was conducted in the absence of any commercial or financial relationships that could be construed as a potential conflict of interest.
